# Preparation, Surface Characterization, and Water Resistance of Silicate and Sol-Silicate Inorganic–Organic Hybrid Dispersion Coatings for Wood

**DOI:** 10.3390/ma14133559

**Published:** 2021-06-25

**Authors:** Arnaud Maxime Cheumani Yona, Jure Žigon, Alexis Ngueteu Kamlo, Matjaž Pavlič, Sebastian Dahle, Marko Petrič

**Affiliations:** 1 Department of Wood Science and Technology, Biotechnical Faculty, University of Ljubljana, Jamnikarjeva ulica 101, SI-1000 Ljubljana, Slovenia; jure.zigon@bf.uni-lj.si (J.Ž.); Matjaz.Pavlic@bf.uni-lj.si (M.P.); Sebastian.Dahle@bf.uni-lj.si (S.D.); marko.petric@bf.uni-lj.si (M.P.); 2 Macromolecular Research Team, Faculty of Science, University of Yaoundé 1, Yaoundé P.O. Box 812, Cameroon; akngueteu@yahoo.fr

**Keywords:** wood, silicate, sol-silicate, coating, roughness, adhesion

## Abstract

The purpose of this study was to comparatively investigate the behavior of silicate and sol-silicate coatings non-modified or modified with an organosilane on wood and on wood pre-coated with silica-mineralized primers. Adhesion strength, morphology, and water permeability and related damages were studied to evaluate the possibility of utilizing such inorganic-based coating systems for durable protection of wood without or with relatively cheap and water-borne primers. Potassium silicate and potassium methylsiliconate aqueous solutions and a colloidal silica were used for the preparation of the coatings. The white coating paints were brushed on beech wood substrates at a rate of 220 g·m^−2^. The coatings exhibited good coverage ability. The pull-off adhesion strength values appeared to be related to pH following a polynomial law. The adhesion strength for the silicate coatings were adequate (above 3 MPa and up to 5 MPa) for wood, whereas the values for the sol-silicates were too low for practical applications. The adhesion values were in general higher for the samples cured in a climate room (23 ± 3 °C and 75 ± 2% relative humidity) than the samples cured in the ambient atmosphere of the laboratory (23 ± 3 °C and 25 ± 5% relative humidity). The presence of microdefects (cracks, holes) was revealed in the coating layers by optical and scanning electron microscopy. The surface roughness parameters assessed by confocal scanning laser microscopy were dependent on the magnification applied for their measurement. The arithmetic average roughness *S_a_* was between 5 µm and 10 µm at magnification 5× and between 2.5 μm and 10 µm at magnification 20×. The maximum peak-to-valley height *S*_z_ confirmed the presence of open pores emerging through the coatings. The open pores constitute free pathways for water ingress through the coatings, and could explain the high water absorption of the coatings including the methysiliconate-containing silicate coating and despite the relatively high water contact angle and low wettability exhibited by this sample. The post-application of a hydrophobizing solution containing hexadecyltrimethoxysilane and dimethyloctadecyl[3-(trimethoxysilyl)propyl]ammonium chloride considerably reduced the water permeability, while application of nanosilica-containing organic primers increased the adhesion for the coatings. Silicate coatings with adhesion great enough and resistance against water damages can be generated on wood even without a primer using low silica-to-alkali ratio binders and an organosilane additive. The sol-silicate coatings appear to be applicable only with a primer. The improvement of the paint formulations to control the formation of microcracks and open pores could be useful to reduce the liquid water permeability and increase durability. Otherwise, the application of a hydrophobizing treatment can be used for this purpose.

## 1. Introduction

Silicon-based inorganic-organic hybrid coatings are interesting alternatives to conventional organic-based coatings for wood protection because they can provide high resistances to scratch and impact, UV-light, heat and fire, and increased durability if properly designed and applied. These coatings have proved to be resilient and performant in both indoors and outdoors applications on mineral substrates and for corrosion protection of metals [1,2,3]. Silicon is the second-most abundant element in the earth’s crust after oxygen and is readily available for processing into various inorganic and inorganic-organic hybrid products with versatile industrial applications. Water glass, colloidal silica, and organosilanes are common products gaining attention and acceptance in adhesive and coating sectors. Water glass and colloidal silica are relatively cheap and environmentally friendly materials with binding and reinforcing properties. The binding ability of water glass has been used in the foundry for the manufacture of mold and core molding sands and in the development of silicate-based coatings and adhesives [1,3,4,5]. The addition of colloidal silica in conventional organic coatings has resulted in improvement of hardness, scratch and corrosion resistance, shear and bonding strengths [6,7]. Colloidal silica (or silica-sol) and organosilane compounds (e.g., (glycidoxypropyl)trimethoxysilane, hexadecyltrimenthoxysilane) have been combined to produce denser thin hybrid film coatings exhibiting better adhesion on various substrates including wood [8,9,10,11,12,13,14].

Silicate and sol-silicate dispersion paints are coating systems using a water glass or a dispersion mixture of a water glass and a colloidal silica as the main binder, mineral fillers, and pigments such as calcium carbonate, zinc oxide, clays, and titanium dioxide [1,3,15,16,17]. Silicate and sol-silica paints are established in the field of mineral substrates and metal protection. A technology builds upon the use of a hydrophobic and mineralized drying oil or alkyd-resin primer has been developed by companies (e.g., Keimfarbein GmbH, Diedorf, Germany; Beeck’sche Farbwerke GmbH, Laichingen, Germany) to exploit the advantages of these coatings in wood. The drying oil and alkyd resins provide good adhesion to the wood while dispersed minerals in the primer layer ensure adhesion with the silicate topcoat. However, the scientific literature on these coatings in the field of wood and data on their use without primers or with other potentially cheap primer systems remains scarce. The pure inorganic silicate or dispersion silicate coatings, as formulated for minerals substrates, are unlikely to perform well and durably on wood and wood materials because of their low flexibility (a propriety inherent to inorganic materials). Wood exhibits higher moisture dimensional changes than mineral substrates and metals, which could cause cracking and debonding of the coatings.

Water glass is an alkaline solution containing a wide range of chemical species from monomeric to oligomeric silicates. Their properties including binding ability vary as a function of molar ratio of silica and alkali SiO_2_/M_2_O, the nature of the alkali metal (M) and mass concentration. Potassium water glass is the most used for coating applications for technical and economic reasons: it can be cured at ambient conditions contrary to sodium water glass, and it is less expensive than lithium water glass. The SiO_2_/M_2_O molar ratio of water glass is in general below 4, but the addition of silica and especially nanosilica (e.g., colloidal silica) increases the SiO_2_/M_2_O molar ratio and yields to the so-called sol-silicate. When nanosilica is appropriately added, a dispersion stable over gelling for an acceptable period can be obtained. The chemical reactions between water glass and colloidal silica change the particle size and their distribution and contribute to the growth of smaller silica particles in the mixture [18]. The sol-silicate paints have exhibited improved resistance to weather (freeze–thaw cycles), adhesion, and strength development compared to silicate coatings [16,17]. Organic polymers and organosilanes can also be utilized to modulate the properties of silicate and sol-silicate coatings. Organic polymers have appeared to be effective admixtures for mechanical reinforcements of silicon-based materials. Flexible silica aerogels and silica coating films have been synthetized from formulations containing organo-trialkoxysilane compounds such as methyltrimethoxysilane [19,20,21,22].

The development of silicate and sol-silicate coatings suitable for wood and wood materials requires a good understanding of the behavior of such coatings at the surface of wood. The key-parameters to control and produce durable and efficient finishes must be defined considering the different silica-to-alkali ratio and various chemical additives that could be utilized. The adhesion of silicate coatings on wood was shown in literature [23,24]. Kazmina et al. [23] reported for the investigated paints adhesion strength between 5 and 6 MPa without data on the physicochemical interactions between the coatings and wood, and their durability; the main objective of their study was the contribution of a magnesium-containing additive on the fire-proofing performances of the silicate coatings. In a previous work from our group [24], some silicate formulations were studied, their adhesion on wood appeared dependent on the composition, poor to moderate and acceptable adhesion strength values were measured. The parameters such as solid content, the addition of coalescent chemicals or compounds interacting with silicate species (e.g., sugar) reduced the penetration of the coatings inside the wood and significantly decreased the adhesion strength [23,24]. The substitution of a part of potassium water glass binder by potassium methylsiliconate water solution in the preparation of silicate coatings for wood substrate showed improvement of the resistance to cracking and chalking but without a considerable reduction of the water permeability [24]. It should be mentioned that the formulations studied in the previous studies [23,24] were pre-formulations without stabilizers or thickeners, and exclusively silicate coatings. To the best of the knowledge of the authors, there is not yet in the literature reports addressing the behavior of fully formulated silicate and sol-silicate coatings on the surface of wood.

The aim of this work was to study the behavior of silicate and sol-silicate coatings non-modified or modified with an organosilane (potassium methylsiliconate) on wood and on wood pre-coated with different mineralized primers. Adhesion strength, morphology, water permeability, and related damages were studied to evaluate the possibility of utilizing such inorganic-based systems for durable protection of wood without or with relatively cheap and water-borne primers. The silicate and sol-silicate coatings were prepared and their physico-chemical properties such as pH, density and viscosity at various shear rates were determined. The coatings were applied on European beech (*Fagus sylvatica* L.) wood by brushing at a rate of 220 g·m^−2^ (110 g·m^−2^ twice). Curing was performed at ambient conditions 23 ± 2 °C and 25 ± 5% RH air relative humidity [RH]) or in a climate room 23 ± 2 °C and 75 ± 2% RH; RH is the relative humidity of the environment. The cured coatings were characterized by using CIELAB color system, pull-adhesion tests, optical microscopy for thickness (magnification 10× and 20×), and the correlation between pH and adhesion were established. Confocal laser scanning microscopy (CLSM) and scanning electron microscopy (SEM) were used for the characterization of surface morphology and interface between the wood and the coatings. The arithmetic average roughness (*S*_a_) and maximum peak to valley roughness (*S*_z_) are described. The coatings were exposed to water and analyzed before and after exposure by attenuated total reflectance-Fourier transform infrared (ATR-FTIR) spectroscopy and SEM coupled with energy dispersive X-ray (EDX) analysis for surface morphology and chemical changes. The influence of application of silica-based organic primers on adhesion or post-application of an hydrophobizing aqueous dispersion on water absorption was also studied.

## 2. Materials and Methods

### 2.1. Materials

European beech wood samples (320 × 80 × 10) mm^3^ (longitudinal × tangential × radial) were used for the experiments. The coatings were applied on longitudinal-tangential surfaces. Silica gel (porosity 60 Å, particle size 63–200 µm), methyltrimethoxysilane (MTMS), 98%), potassium hydroxide (90%), colloidal silica Ludox^®^ AS (40 wt % suspension in water), titanium(IV) oxide (99.5%, 21 nm mean particle size), hydroxyethylcellulose (HEC), and silicon antifoam (SaF, 30% in water, emulsion), hexadecyltrimethoxysilane (HDTMS, technical, ≥85%) and dimethyloctadecyl[3-(trimethoxysilyl)propyl]ammonium chloride (DTSACl, 42 wt % in methanol), poly(vinyl alcohol) fully hydrolyzed (P1763) were purchased from Sigma Aldrich Chemie GmbH, Steinheim, Germany. Domemul SA 9263 (styrene-acrylic emulsion, non-volatile matter 39–41%, pH 8–8.5, viscosity at 23 °C 20–350 mPa·s) (Sty-Acr) was provided by Helios TBLUS (Količevo, Slovenia). Zinc oxide (≥99%, Honeywell, Seelze, Germany), talcum (98%, Roth, Karlsruhe, Germany), precipitated calcium carbonate (Fisher Chemical, Loughborough, UK/Acros Organics, Geel, Belgium) were ground manually in a porcelain mortar for 30 min each to reduce the particle sizes and aggregates. Stabilizers (Betolin Q40 (BQ40) and Betolin A11 (BA11)), dispersant (Betolin D20(BD20)) were kindly provided by Wöllner GmbH (Ludwigshafen, Germany). Demineralized water was used. A polyvinylacetate-based adhesive (MEKOL) from Mitol Tovarna lepil d.o.o. (Sežana, Slovenia) (denoted PVAc) and one component waterborne acrylate-polyurethane coating Akzent from Stauf Klebstoffwerk GmbH (Wilnsdorf, Germany) (denoted PU-Ac) were used in the preparation of mineralized primers. Dextrin (DEXT) was purchased from Roth (Karlsruhe, Germany).

### 2.2. Preparation of the Coatings

The recipe for the preparation of the coatings, shown in Table 1, was established based on literature [15,23] and previous works carried-out by our group [24].

The binders were obtained by mixing a potassium water glass (SiO_2_/K_2_O molar ratio 3.2, theoretical solid content 35%), a potassium methylsiliconate aqueous solution (MTMS/K_2_O molar ratio 3.2, theoretical solid content around 28.5%), and colloidal silica Ludox^®^ AS 40. In a typical experimental procedure, potassium water glass was obtained by adding slowly dried silica gel (100.00 g) to an aqueous solution of potassium hydroxide (58.54 g of KOH in 306.30 g of water), the mixture was slightly heated under magnetic stirring until complete dissolution-reaction of the solid silica gel according to reaction Equation (1). The prepared potassium water glass was stable over time and its pH was 12.13. The potassium methylsiliconate solution was obtained by reacting MTMS (50 g) with an aqueous solution (14.71 of KOH in 120.17 g of water). Monomeric and oligomeric methysiliconate species are formed though hydrolysis of alkoxy groups, condensation between silanols and neutralization of end-silanol groups according to reaction Equations (2)–(4). The pH of the methylsiliconate solution was initially 13.16.

(1) $$n{\mathrm{SiO}}_{2}+2\mathrm{KOH}\;+(m-1)H_{2}O\;\to {\;K}_{2}O\cdot n{\mathrm{SiO}}_{2}\cdot mH_{2}O\;$$

Mixing ratios for the preparation of the different binders are reported in Table 2 with designation of the corresponding coatings. SW1 and SW2 were dispersion silicates while SW3, SW4 and SW5 were sol-silicate paints.

(2) $$\mathrm{MeSi}{\left(\mathrm{OMe}\right)}_{3\;}+3H_{2}O\;\to \;\mathrm{MeSi}{\left(\mathrm{OH}\right)}_{3}+3\mathrm{MeOH}\;$$

(3) $$=Si\left(Me\right)\;-\;OH+HO-\;\left(Me\right)Si=\;\;\to \;\;=Si\left(Me\right)\;-\;O-\;\left(Me\right)Si=\;+\;H_{2}O\;\;\;$$

(4) $$=Si\left(Me\right)\;-\;OH+\;KOH\;\;\;\;\to \;\;\;=Si\left(Me\right)\;-\;O^{-}K^{+}+\;H_{2}O\;$$

In a typical experimental procedure utilized for the preparation of the coatings, the stabilizers (BQ40 (Betolin Q40) and BA11(Betolin A11)), the dispersant BD20 (Betolin D20), the thickener HEC (Hydroxyethylcellulose) and the SaF (silicon antifoam) and water were pre-mixed in a polypropylene cup and homogenised for 12 h under occasional stirring for complete dissolution of HEC. Styrene-acrylic dispersion was then added, followed by solid additives (calcium carbonate, talc, zinc oxide, titanium dioxide) while stirring with a glass rod for 3 min. The liquid binder was finally added, and the mixture stirred using an IKa^®^ T25 digital ultra-Turrax^®^ (Staufen, Germany) at 3400, 5000, and 8000 rpm for 2, 2, and 3 min, respectively. The so-obtained coatings were stored for 24 h for maturation before applying on wood.

### 2.3. Application of the Coatings on the Wood Substrate

The surface of the wood was sanded with a 120-grit sandpaper and dusted off with compressed air. The coatings were applied at a rate of 220 g·m^−2^, in two steps of 110 g·m^−2^ spaced by 12 h. The application was performed manually by brushing with a brush No. 20. Two samples of coated wood were prepared for each coating, one stored in the indoor ambient condition (AC) which is generally dried due to heating during winter 23 ± 2 °C and 25 ± 5% RH, and the second in a climate room (CR) containing a saturated solution of sodium chloride 23 ± 2 °C and 75 ± 2% RH). The coated samples were kept for curing for two weeks (14 days) before characterization. The samples placed in a CR were removed after 12 days and kept in ambient condition for drying.

The coatings were also applied on the wood surface pre-coated with different mineralized primer formulations. The primers were mineralized with the colloidal silica Ludox^®^ AS 40. PU-Ac + SiO_2_ primer was obtained by mixing in a weight ratio two parts of the PU-Ac coatings with one part of colloidal silica, PVAc + SiO_2_ primer by mixing in a weight ratio two part of the PVAc with one part of colloidal silica and one part of water. PVA + SiO_2_ and DEXT + SiO_2_ were obtained by mixing 50 g of 10 wt % aqueous PVA or dextrin with 6.25 g colloidal silica. The primers were applied at a rate of 120 g·m^−2^ and the silicate or sol-silicate topcoats were applied at 150 g·m^−2^.

The influence on water absorption during the application of a hydrophobizing agent at the surface of some coatings was also studied. The hydrophobizing agent was prepared by mixing 8 g of HDTMS, 2 g of DTSACl solution, and 90 g of water and stirring for 6 h to obtain an aqueous dispersion. The hydrophobizing agent was brushed at the surface of the coatings at a rate of 100 g·m^−2^.

### 2.4. Characterization of the Coatings

#### 2.4.1. pH and Relative Density

pH was measured with a Mettler-Toledo pH-meter (Mettler Toledo GmbH, Greifensee, Switzerland) equipped with an InLab^®^ Expert Pro-ISM sensor (Mettler Toledo AG, Schwerzenbach, Switzerland). The pH-meter was pre-calibrated before each set of experiments. The density was determined with a 25 mL glass-pycnometer. The relative density was determined as the mass of the sample divided by the mass of distilled water filling the same pycnometer.

#### 2.4.2. Shear Viscosity

The shear viscosities were measured using an ARES G2 rheometer (TA instruments, New Castle, DE, USA) using two plate parallel geometry. Both plates (steel plates) were 25 mm in diameter, and the gap was fixed between 0.9 mm and 1 mm. Flow ramp tests were performed at a shear rate from 0 to 1000 s^−1^ (forward) and 1000 s^−1^ to 0 (reverse) at a temperature of 25 °C controlled by an air flow. At least three replicates were performed for each sample and the results are mean values of the replicates and the viscosity values were read from reverse flow curves.

#### 2.4.3. CIELAB Color Measurements

The CIELAB color parameters, L*, a* and b*, were measured with a spectrophotometer X-Rite SP62 (Grand Rapids, MI, USA) equipped with the D65 type of light source.

#### 2.4.4. Adhesion Strength

Adhesion strength values were determined by pull-off tests according to the standard ISO 4624-2016 [25]. Aluminum dollies (20 mm in diameter) were glued on the surface of the coatings using a 2-component polyurethane adhesive and allowed to cure for 24 h. The coating around the dollies was carefully cleaned down to the substrate to isolate the glued zone from the rest of the coating layer. The tensile stress (adhesion strength) applied to peel off the coating from wood surface was measured by using a Defelsko Positest^®^ Adhesion tester (Defelsko instruments corporation, Ogdensburg, NY, USA). The failure was mainly of adhesive type. Three replicates were performed for each sample and the results are mean values of the replicates.

#### 2.4.5. Surface Morphology

The surface roughness of the coatings was studied using a confocal laser scanning microscope (Olympus LEXT OLS5000, Olympus Corporation, Tokyo, Japan) with a MPLFLN5× (numerical aperture 0.15, working distance 20 mm), MPLFLN10xLEXT (numerical aperture 0.3, working distance 10.4 mm)**,** and LMPLFN20xLEXT (numerical aperture 0.45, working distance 6.5 mm) objectives. The microscope is equipped with a 405 nm violet laser, which enables a lateral resolution of down to 0.12 µm. The 2D CSLM micrographs of the fracture surfaces between wood and the coatings were used for the determination of the thickness of the various coatings; the values were average of at least 10 measurements performed on various samples and at different locations. The surfaces of the coatings and their interfaces with the wood were also analyzed by SEM-EDX. The micrographs were taken at a 20 kV voltage and a pressure of 50 Pa using a large field (LFD) detector in a Quanta 250 scanning electron microscope (FEI Company, Hillsboro, OR, USA) at working distances between 7 and 11 mm at magnification 150× and 300×. The analyzed interfaces were fracture surfaces obtained by splitting the coated wood samples in the longitudinal direction.

#### 2.4.6. Contact Angle and Water Absorption Measurements

The contact angle between a water droplet and the coating’s surface was measured with an optical goniomether Theta (Biolin scientific Oy, Espoo, Finland) equipped with the software OneAttension version 2.4 (r4931) from the same company. A 5-µL droplet was deposited at the surface of a coating and contact angle followed for 60 s. Three to four trials were performed for each sample. Water absorption tests were carried out according to EN 927-5 [26] with a modification (different sample sizes, no pre-conditioning). The samples of approx. (100 × 55) mm^2^ in size were cut from coated wood and the uncoated surfaces were covered with paraffin. Each test specimen was exposed to demineralized water (300 mL) floating on the surface of the water with the investigated coated side facing down. The mass increase associated to water uptake was followed for 72 h.

#### 2.4.7. ATR-FTIR

Attenuated total reflection-infrared (ATR FTIR) spectroscopy was performed using a Spectrum Two (PerkinElmer Inc., Waltham, MA, USA) ATR FTIR spectrometer, with a LiTaO_3_ detector in the absorbance mode. The spectra were corrected for background noise and 16 scans per sample were collected at a wavelength from 400 cm^−1^ to 4000 cm^−1^ at a resolution of 0.5 cm^−1^.

## 3. Results and Discussion

### 3.1. Physicochemical Properties of the Fresh Coatings

The pH and relative densities of the coatings are reported in the Table 3.

The coatings exhibited different pH values; the pH of the silicate coatings being higher than the ones of sol-silicates. An increase of the silica-to-alkali ratio reduces the pH of the mixture. The pH of the coatings dropped after 24 h of maturation as can be seen with the differences in the values of the pH within an hour of the preparation (pH 1 h) and pH after 24 h. The pH change was more important in the sol-silicate coatings suggesting a low chemical stability of these coatings. The chemical stability of the coatings depends on the mutual reactions between the components. The chemical reactions between silicate species, hydroxyl ions and polyvalent metal cations (e.g., Ca^2+^, Zn^2+^) could occur at the interfaces of the mineral fillers depleting the hydroxyl ions in the paint solution. The decrease in pH causes polymerization of silicate species which could increase the viscosity and lead to gelation of the paint. The change of the pH with time showed that additional care must be taken to limit the reactions by adjustment of stabilizer’s nature and contents for long pot life. The relative density of the coatings was between 1.40 and 1.42 kg m^−3^.

The storage, leveling, and sagging, and application properties of a coating are rheology-dependent and can be determined by the rheological behavior such as flow ramp. The viscosities at some defined shear rates of the coatings obtained from the backward flow ramp curves (viscosity as a function of shear rate) are shown in Table 3. The viscosity of the coatings at 0.1 s^−1^ were above 50 Pa·s, suggesting a good resistance to settling during storage. In fact, silicate coatings behave like a solid gel at rest displaying high viscosities at low shear rates, viscosity that drops drastically as the shear rate increases as reported previously [24]. From the rheological studies of some waterborne paints, it was established that for drying with a good levelling and minimum sagging, the viscosity at shear rate of 1 s^−1^ should be between 5 Pa·s and 10 Pa·s [27]. The viscosity of the coatings was higher to this range. The shear rate range between 10^3^ s^−1^ and 10^6^ s^−1^ are associated with the common brushing (or rolling) application mode, and the viscosity for an easy brush application without excessive drag is said to be between 0.1 and 0.3 Pa·s [27,28]. The viscosity values obtained at 10^3^ s^−1^ were between this range for the coatings investigated, except at SW3 and SW4. The optimization of rheological properties of these coatings is not in the scope of this work and will be performed elsewhere.

### 3.2. Surface Appearance, Thickness, and Adhesion of the Coatings on Wood

The CIELAB color parameters of the cured coatings at the surface of beech wood are shown in Table 4. A photograph of the coatings is shown in the Supplementary Material (Figure S1).

**Figure 1:**
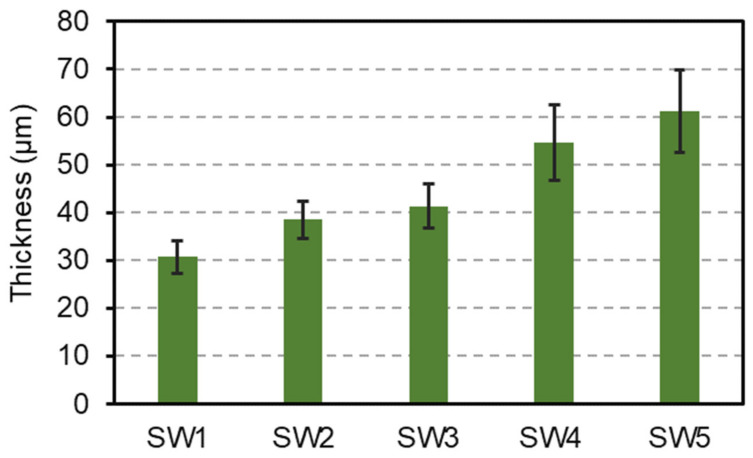
Thickness of the cured coating layers.

The surface of wood was well covered by the coatings. The wood features were no longer perceptible. In the CIELAB color system, L* indicates the lightness of the sample with values from 0 (black) to 100 (white), whereas the chromatic coordinates a* and b* represent green-red and blue-yellow axes, respectively, with negative values corresponding to green and blue and positive values for red and yellow [29]. An increase of lightness (L* values) was observed for all the coatings compared to uncoated wood resulting from the whitening of the surface. The sol-silicate coatings (SW3 to SW5) yielded higher L* values than the silicates (SW1 and SW2). SW1 showed the smallest lightness value, the addition of potassium methylsiliconate in SW2, SW4, and SW5 increased slightly the lightness of the coatings (SW4 and SW5 compared with SW3). The yellow-red color of the wood was almost not observable when wood was coated with all the formulations as shown by the values of a* and b* close to zero (between −1 and +1). The sol-silicates showed generally a better coverage ability and whiteness than the silicate coatings at the wood surface. The mean thickness values of the coatings determined by optical microscopy can also explain the results (see Figure 1). The lightness increased with the coating’s thickness since a higher thickness of the layer hides more of the surface beneath. The state of advancement of the conversion of silicon compounds in the binders could also contribute to the color difference between the silicate and sol-silicate coatings. A water glass leads to a transparent layer on drying whereas silica-sol (colloidal silica) produce a white layer on drying due to aggregation of the silica particles. It can take many months for a transparent layer of water glass to whiten due to the formation of silica through the reaction between silicate species, carbon dioxide from air, and water in the pores.

**Figure 2:**
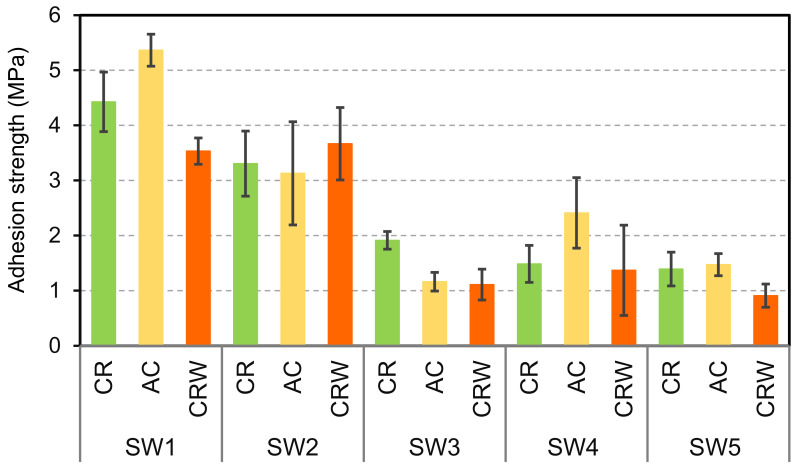
Adhesion strength of the coatings at the surface of beech wood. CR: curing in climate room 23 ± 2 °C and 75 ± 2%RH; AC: curing in ambient conditions 23 ± 2 °C and 25 ± 5%RH; and CRW: coatings cured in climate room, exposed to liquid water for three days, and dried in ambient conditions for two weeks.

The adhesion strength values of the coatings at the surface of beech wood are shown in Figure 2.

SW1 and SW2 exhibited relatively good adhesion, adhesion strength between 3 to 5 MPa, while the adhesion of sol-silicates SW3-SW5 was lower. The coatings were cured in climate room (CR) or in ambient conditions (AC). The adhesion values were globally slightly higher for CR curing than AC curing, except for SW1. The curing of these coating systems required moisture and carbon dioxide from air [1]. The RH in the ambient condition of the laboratory was low around 23 ± 2% when the experiments were performed and could yield too fast drying. The low moisture content in the materials could reduce the dissolution of carbon dioxide from air or also the interactions between the reactive mineral fillers (calcium carbonate, zinc oxide) and the silicate binding species. However, in CR at 75 ± 2% RH, moisture in wood and the coatings are relatively higher than in AC, but the supply of carbon dioxide is reduced. A closed CR was used in this study. The extent and rate of curing of some silicate-based coatings were found to be related to the RH of the atmosphere to which the coatings are exposed, and curing at lower RH (e.g., 40% RH) was unlikely to achieve satisfactory curing state even after prolonged cure [30]. The further characterizations that are shown below and in the next sections were mainly performed on the coating samples cured in CR.

**Figure 3:**
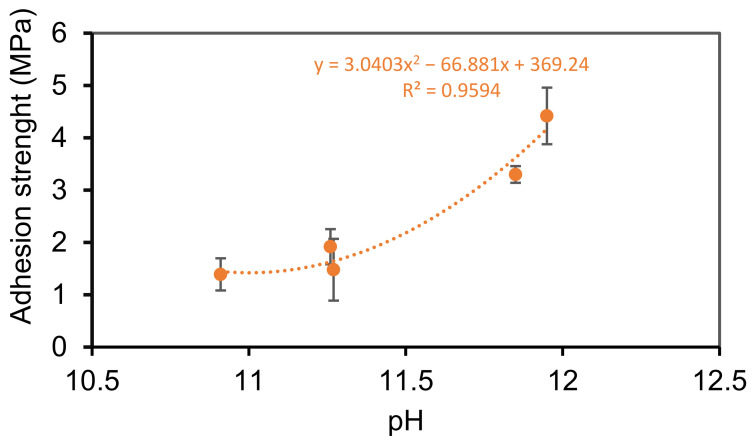
Correlation between pH and adhesion strength.

The difference in the adhesion values of the coatings was primarily related to the ability of the coatings to penetrate into the wood and formation of mechanical anchorages at the interface with wood. The difference in the coating thickness was attributed to difference in the amount of the coatings infiltrated within the wood considering that comparable rate was applied for each coating (around 220 g·m^−2^) although drying-shrinkage can contribute to this phenomenon. The analyses of the results showed that the adhesion strength of the coatings increased with the increasing pH value of the liquid coatings (see Figure 3).

The linear correlation coefficient R^2^ between the adhesion values and the pH was relatively low (R^2^ = 0.884) and increased for a 2nd polynomial law. The adhesion seemed to be related to the pH following a polynomial law. The pH probably defined the penetration of the coatings within the wood. The penetration of silicate and sol-silicate products inside the wood have appeared to be more related to the pH than to other properties such as viscosity [31,32]. An increase of the pH decreases the particles sizes and favors penetration through wood pores [31], and the pH must be sufficiently high to reduce the impact of wood acidity on the property of the impregnation chemicals. At the contact of the wood surfaces that are in general slightly acidic, the pH can be dropped in such a way it causes aggregation of the silica particles of the sol-silicate binders, blocking pore entries for capillary permeability. The pH of the sol-silicate coatings was below 11.3.

### 3.3. Surface Morphology of the Cured Coatings

**Figure 4:**
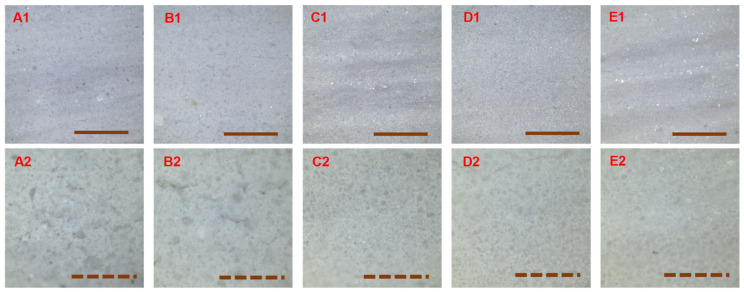
2D micrographs of the surface of the cured coatings at magnifications 5× (**A1**–**E1**) and 20× (**A2**–**E2**): SW1 (**A**), SW2 (**B**), SW3 (**C**), SW4 (**D**), and SW5 (**E**) (the solid line scale bars represent 1 mm and the dash line scale bars 300 µm).

Figure 4 shows the 2D micrographs of the surfaces of the cured coatings at the magnifications 5× and 20×. The surfaces looked quite uniform at magnification 5× with few dispersed and discontinued fissures, but as the magnification was increased to 20×, the presence of holes and microcracks was more revealed. The microcracks were slightly more important at the surface of the coatings containing potassium methylsiliconate (SW2, SW4, and SW5).

**Figure 5:**
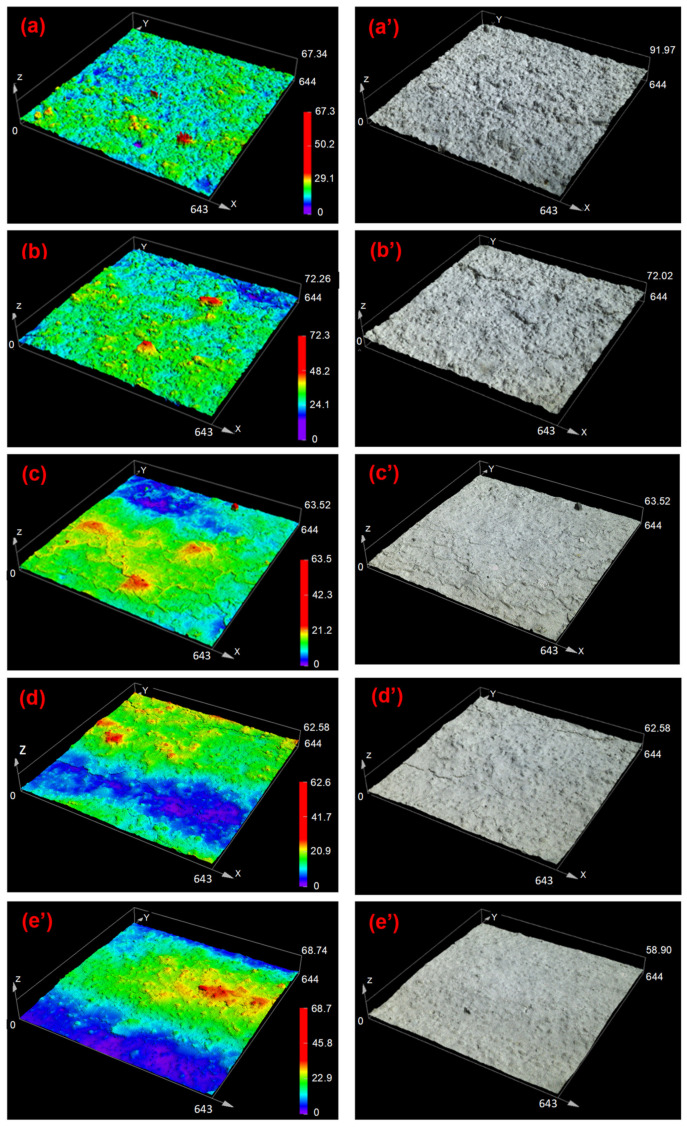
3D micrographs of coatings recorded by CLSM at magnification 20× (size of analyzed spots [640 × 640] µm^2^): SW1 (**a**,**a’**), SW2 (**b**,**b’**), SW3 (**c**,**c’**), SW4 (**d**, **d’**), and SW5 (**e**,**e’**).

The 3D micrographs of the coatings’ surfaces were recorded at magnification 5× and magnification 20×. The color images with height data and laser images obtained at magnification 20× and shown in Figure 5 are displaying more the roughness features of the surfaces. Some images recorded at magnification 5× are shown in the Supplementary Material (Figure S2). The results for the silicates SW1 and SW2 showed peaks and valley randomly distributed at the surface. The surface irregularities ensued from low film-forming ability of the coatings, an inherent property to inorganic products. The peaks and valleys were larger for sol-silicates, giving a flatter wave-like appearance to these surfaces.

**Figure 6:**
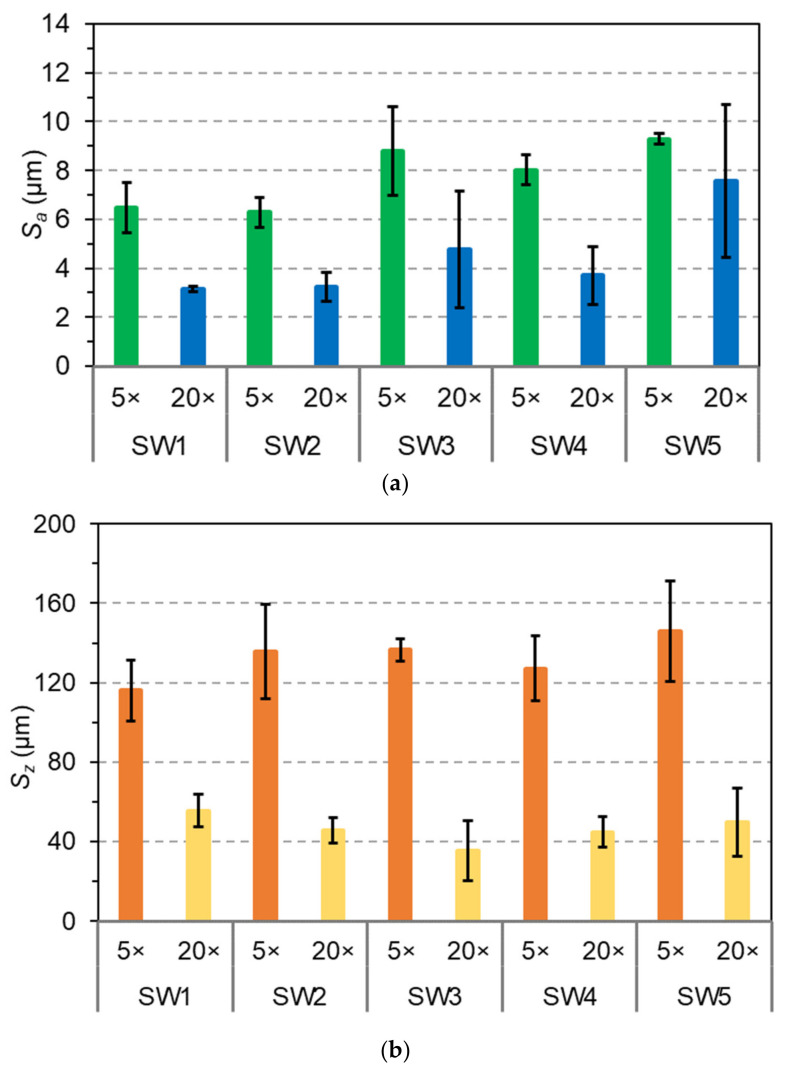
Surface roughness parameters of the coatings (**a**) arithmetic average *S*_a_ and (**b**) maximum peak-to-valley height *S*_z_.

The arithmetic average roughness *S*_a_ and maximum peak-to-valley height *S*_z_ as determined by CLSM at magnification 5× and 20× are shown in Figure 6.

The roughness parameters calculated from the 3D micrographs varied with the magnification applied for their measurement and were higher at low magnification. The increase of the magnification reduced the analyzed area and probably consequently the impact of surface defects such as brush drags. The length-scale dependence of surface roughness parameters measured by CLSM has been demonstrated in literature [33]. Among the parameters, arithmetic average roughness *S*_a_ (or *R*_a_) is commonly chosen to express to surface roughness of materials. A larger *S*_a_ value indicates a higher roughness [34]. The *S*_a_ values at magnification 5× was between 5 and 7.5 µm for silicate coatings (SW1 and SW2) and between 7 and 10 µm for the sol-silicate coatings (SW3–SW5). The smallest value of *S*_a_ for this magnification were obtained with SW2. The *S*_a_ values were reduced almost by half when measured at magnification 20×, but a similar trend was observed between the coating samples as at magnification 5×. The results suggested that the sol-silicates were globally slightly rougher than the silicate coatings for the formulations investigated. This range of surface roughness is usually characteristic of matt and non-film forming inorganic surfaces. Similar higher *S*_a_ values were reported for silicate coatings on concrete and for inorganic dental porcelain [35,36]. Surface roughness of organic film-forming coatings can be below 1 µm [33]. However, the human’s perception of a rough surface depends on the distribution of peaks and valleys. A rough surface can be perceived as smooth when there are some regularities in peak-to-valley distributions or the irregular peaks stacking together to form larger crepes as reported by Ismail et al. [37].

The *S*_z_ parameter was differently affected by the coatings, and the lowest values were obtained with SW1 at magnification 5× and with SW3 at magnification 20×. The *S*_z_ values were between 100 and 170 µm at magnification 5× and between 20 and 70 µm at magnification 20×. The *S*_z_ values were higher than the measured thickness of the coatings, especially at magnification 5×, suggesting for the coatings an open porous structure with some pores passing through the coating layer.

**Figure 7:**
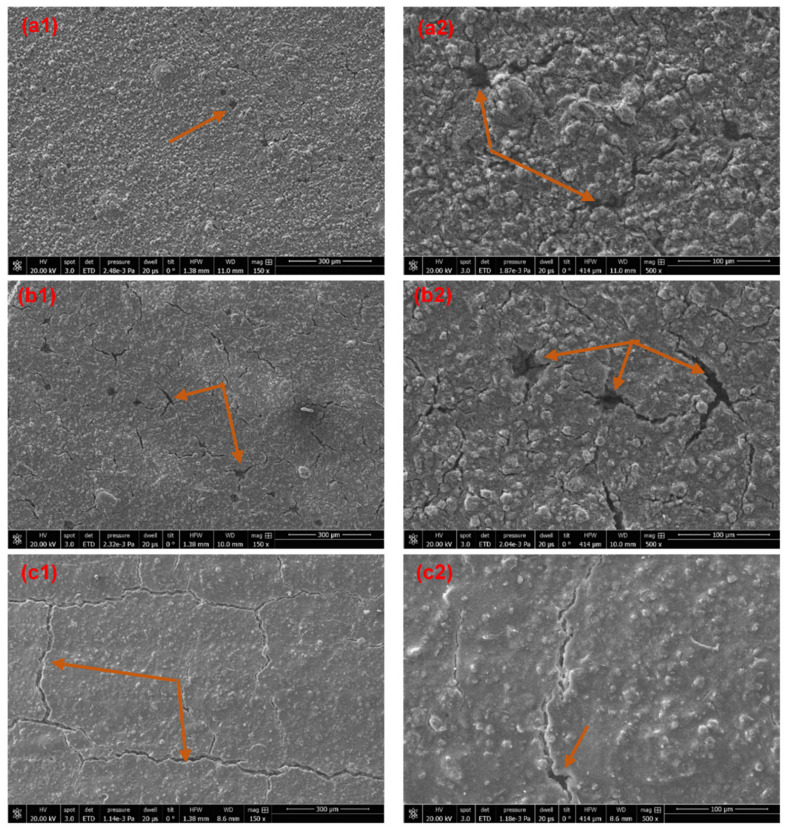
SEM Micrographs of cured SW1 (**a1**,**a2**), SW2 (**b1**,**b2**), and SW5 (**c1**,**c2**) (the arrows indicate cracks and holes).

The presence of microscopic holes and cracks were confirmed by SEM images (Figure 7). This characterization was carried out only for SW1, SW2, and SW5 as selected representant of the sol-silicate coatings).

A continuous network of microcracks was observed in the micrograph of the sol-silicate coating SW5, while the microcracks remained irregular and not connected in the silicates SW1 and SW2. The formation of the microcracks can be explained by rapid drying of the coating layer especially with the addition of methylsiliconate which is basically a hydrophobizing organosilane. The microcracks appeared slightly more important in coating samples cured at ambient conditions (AC) on CLSM micrographs and the values of *S_z_* tended to be higher compared to CR cured coatings; some comparative CLSM images and *S_a_* and *S_z_* values are shown in the Supplementary Material (Figures S3 and S4). The emergence of shrinkage cracks during drying and curing is widespread in inorganic materials. Concrete is a well-studied example [38,39,40]. Large continuous macroscopically visible cracks were reported in activated fly ash slag/sodium silicate coatings brushed on concrete substrates and were reduced by a carboxymethyl starch admixture acting by retaining water in the coatings during curing [41]. Organosilanes such as MTMS and HDTMS are used in the preparation of crack-free and flexible silica aerogels [42]. The addition of methysiliconate that was expected to increase to the flexibility of the coatings and reduce cracking failed in the protection of the coatings against this phenomenon, it even contributed to the increase of cracks. The reduced size of the coating layer and hydrophobicity of the additive favored the evaporation of water, leading probably to materials with low curing advancement.

### 3.4. Contact Angle and Water Absorption

**Figure 8:**
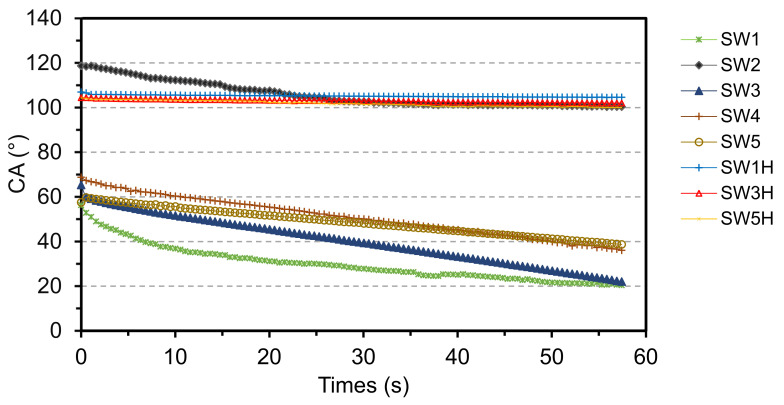
Dynamic wettability of the silicate coatings at the surface of wood. SW1H, SW2H, and SW5H are SW1, SW2, and SW3 post-treated the hydrophobizing dispersion (HDTMS/DTSACl).

The evolution of contact angle between water droplets and the coated surfaces as a function of time are shown in Figure 8.

**Figure 9:**
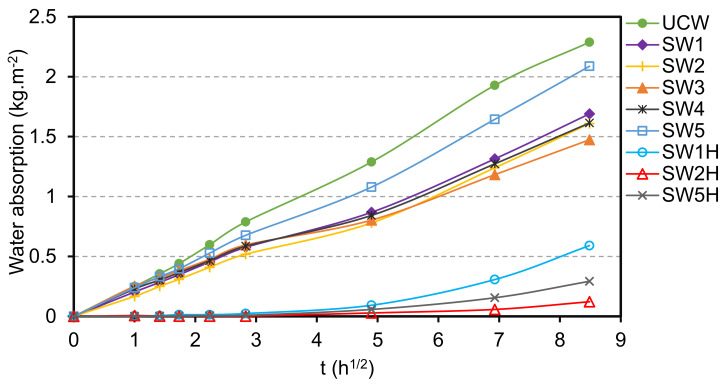
Water absorption curves of the coatings at the surface of wood. UCW is uncoated wood and SW1H, SW2H, and SW5H are SW1, SW2, and SW3 post-treated the hydrophobizing dispersion (HDTMS/DTSACl).

The coatings prepared without potassium methysiliconate (SW1 and SW3) exhibited initial contact angles between 55° and 65° which decreased to approximately 20° after 60 s. The addition of methylsiliconate in sol-silicate coatings (SW4 and SW5) has not considerably changed the hydrophilic nature of the materials, as their wettability was only slightly reduced. In contrast, SW2 showed a marked hydrophobicity with contact angle values above 100° even after 60 s. However, the water absorption profile of SW2 was not different from the ones of the other coatings, despite of its displayed hydrophobicity (See Figure 9).

**Figure 10:**
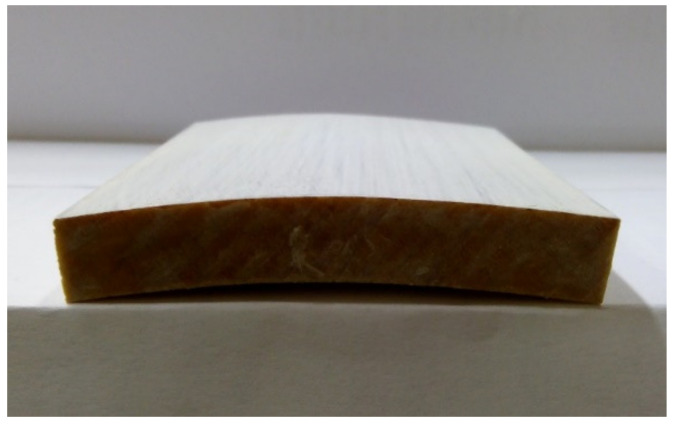
Bow deformation of a test specimen (SW2) after three-day stay in water.

The water absorption of the samples was attributed to the presence of microcracks and open pores that constitute pathways for infiltration of water. As already reported previously [24], SW2 was not dramatically affected by absorbed water and ensuing swelling-deformation of the samples, while visible cracks were perceptible on the other coatings. Beech wood is quite sensitive to water and readily undergoes cupping or bowing in water (see Figure 10).

**Figure 11:**
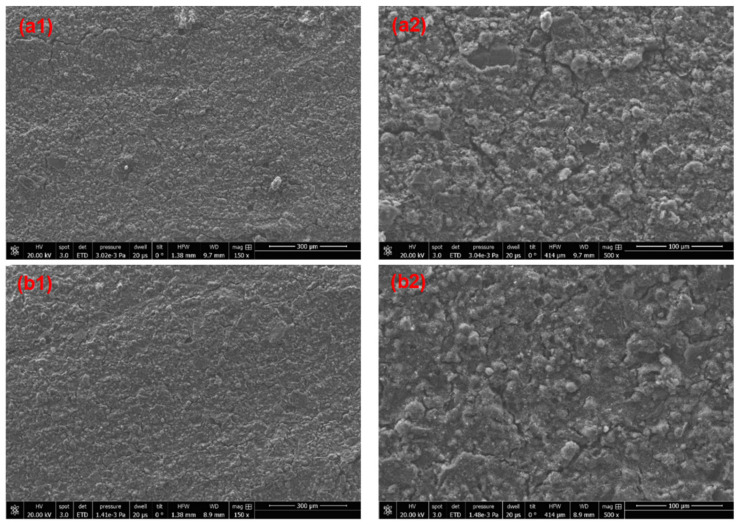
SEM micrographs of the surfaces of the silicate coatings SW1 and SW2 before (**a1**,**b1**) and SW2 (**a2**,**b2**) after exposure to wate.

**Figure 12:**
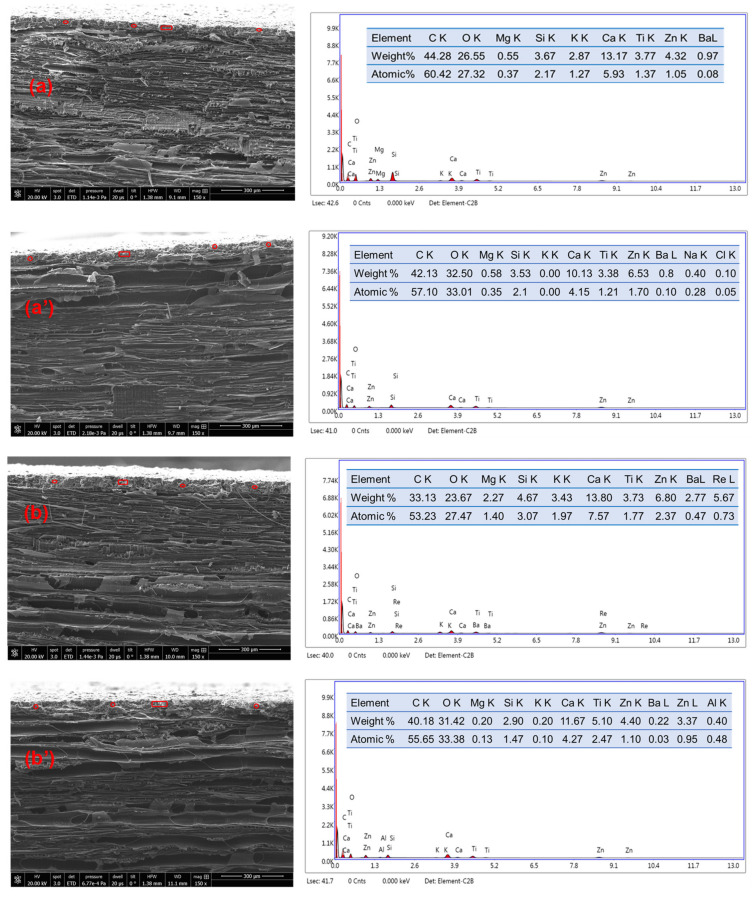
EDX analyses of the coatings SW1 (**a**,**a’**) and SW2 (**b**,**b’**) before (**a**,**b**) and after (**a’**,**b’**) water exposure. Analyses were performed at different area of the fractured surfaces following to the encirclements.

The adhesion of the coatings immersed in water for three days and dried for two weeks in ambient air was measured. The results are displayed in Figure 2 for comparison with adhesion before water exposure (see CRW coatings cured in climate room, exposed to water for three days and dried in ambient conditions for two weeks). The results showed a decrease of the adhesion for all the coatings, except for SW2. Instead, the adhesion of SW2 was slightly increased from 3.31 ± 0.59 MPa to 3.67 ± 0.66 MPa. This improvement was attributed to progression of the curing process of the sample during drying, whereas the decrease of the adhesion strength was related to cracking and debonding after exposure to water. The SEM micrographs of SW1 and SW2 after exposure to water were compared (see Figure 11); the sol-silicates coatings were not further characterized because of significant debonding. The results revealed less holes and cracks in SW2, while the cracks were increased at SW1 all in comparison with micrographs before exposure previously shown in Figure 7. Semi-quantitative EDX analyses performed inside the coating layers (see Figure 12) showed mainly only a considerable decrease of the amount of the potassium element.

The potassium ions from the binder form water-soluble compounds such potassium carbonate in the cured coating. These compounds were completely leached by water in SW1; potassium element was no longer detected. Residual potassium was still observed in SW2 suggesting there were some regions in the coating less accessible to water. The silicon element percentage was less affected by water leaching showing that the water-soluble silicate species were converted into insoluble compounds. The relative percentage of carbon and oxygen elements were increased probably due to further carbonation of coatings after water exposure.

**Figure 13:**
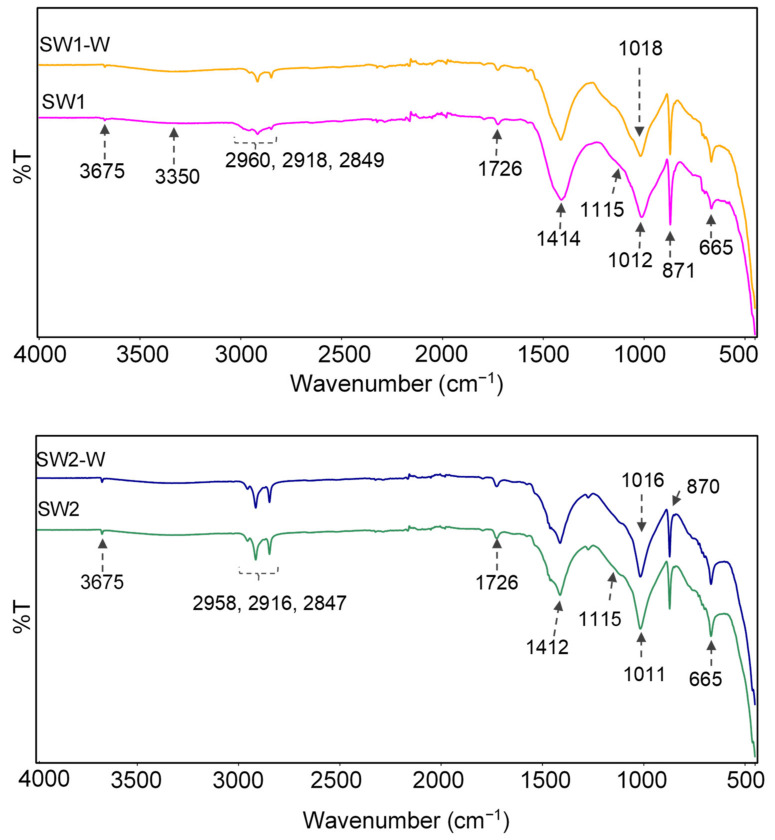
ATR-FTIR spectra of the coatings before (SW1 and SW2) and after (SW1-W and SW2-W) exposure to liquid water.

ATR-FTIR spectra of SW1 and SW2 before and after exposure to water are reported in Figure 13.

The bands at 3675 cm^−1^ and 3456 cm^−1^ occurred from the stretching vibrations of hydroxyl groups of silanol (Si–OH) in silicate products, in talc (Mg–OH) and in absorbed water, mutually H–bonded [43,44,45]. The bands between 2960 cm^−1^ and 2840 cm^−1^ were attributed to vibrations of the C–H bonds in methylene and methyl groups of the various organic additives. The carbonate groups mainly from calcium carbonate gave bands at 1412–1414 cm^−1^ and 871–872 cm^−1^ corresponding to asymmetric stretching and out-of-plane bending of CO_3_^2−^ [46,47]. The Si−O vibrations in silicate compounds provided various bands in the region 1200–900 cm^−1^ with wavenumbers decreasing with the cross-linking degree of the silicon, i.e., highly cross-linked silicon Q4 in closed cage appearing around 1120 cm^−1^, silicon in amorphous gel at 1080 cm^−1^, and silicon in short polymer chain and silicate monomers between 1020–1000 cm^−1^. The shift of the main Si–O bands from 1011–1012 cm^−1^ to 1016–1018 cm^−1^ after water exposure was attributed to the progression of the curing reaction that led to increased polymerization and cross-linking of silicate compounds [43,44,45]. The intensities of the bands in the Si−O vibration region remained practically unchanged, confirming the low leaching of the silicates associated to their conversion into insoluble compounds.

### 3.5. Improvement of Water Resistance and Adhesion

This study was carried out on the samples SW1, SW2, and SW5. The surfaces of the coatings were treated with an aqueous dispersion containing HDMTS and DTSACl and water absorption of the treated samples was studied. The results are shown in Figure 8 and Figure 9 (SW1H, SW3H, and SW5H designations). The hydrophobization treatment reduced the water uptake for all the coatings. The treated samples started to absorb water after two days in water and the amount of water absorbed after the three-day immersion was between 0.12–0.59 kg·m^−2^ compared to 1.5–2.1 kg·m^−2^ for untreated coatings. This effect was not observed when the post-treatment was performed with an aqueous solution of potassium methylsiliconate (10 wt %, MTMS/K_2_O molar ratio 2.8) (results not shown), because the curing conditions were probably not sufficient to achieve the hydrophobization with this solution.

### 3.6. Improvement of the Adhesion of the Coatings

**Figure 14:**
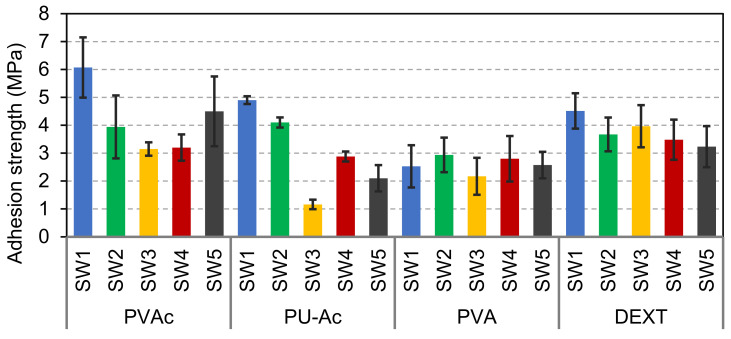
Adhesion strength of the coatings applied on wood pre-coated with PVAc- or PU-Ac, PVA, and DEXT-silica primers.

Primers and intermediates are widespread in the coating sector. PVAc, PU-Ac, PVA, and dextrin mineralized with colloidal silica (Ludox^®^ AS 40%) were applied as primers to the wood surface before application of the silicate and sol-silicate coatings. The adhesion results are shown in Figure 14.

The adhesion of the coatings was improved in almost all cases. Adhesion values above 6 MPa was obtained with SW1 and PVAc-silica primer, while PVA-silica primer gave a reduction of the adhesion of this coating. The PVA-silica was probably sensitive to, and degraded by the alkalinity of the coatings leading to failure of adhesion. The adhesion values of sol-silicate coatings were increased to level that could be acceptable for their application at the wood surface (above 3 MPa) with PVAc and DEXT-based primers.

### 3.7. Discussion

The main difference between the coatings explored in this study were the binders. The binders were silicate solutions or sol-silicate dispersions of different silica-to-alkali ratio. The physicochemical and rheological properties of the coatings were considerably affected by this difference, sol-silicate leading to lower pH and lower viscosity products. These coatings exhibited good coverage abilities at the surface of wood at an acceptable rate of application (220 g∙m^−2^). The sol-silicate showed higher lightness values corresponding to better whitening of the surface and appearance of the white paints. However, the difference in the surface appearance could be due to the thickness of the investigated coatings. The adhesion strength values of the silicate coatings were significantly higher than the adhesion of sol-silicates coatings of the study. Sol-silicate coatings obtained by addition of reduced amount of nanosilica (only slight increase in silica-to-alkali ratio in comparison with the silicate binder) have in the literature shown better adhesion than the silicate counterparts on mineral substrates [16,48]. Maybe the silica-to-alkali ratio was too large in this study and can be optimized by modifying the ratio between potassium silicate, methylsiliconate, or their mixture thereof with colloidal silica. Adhesion strengths even lower than the values found in this study are reported for such coatings at surface of the conventional concrete or steel substrates (1–3 MPa) [49,50], nevertheless the susceptibility of wood to high dimensional changes is a major issue for the stability of the coatings in the environment, especially in rain and moisture conditions of exterior applications. The debonding of the coating layers was already perceptible in the borders of sawing line when sol-silicate coated wood samples were cut indicating the low performances of these coatings.

Considering that the coatings were applied at the same rate per square meter, approximately the same range of thickness was expected. However, a noticeable difference in the measured values was observed. Two phenomena can explain these disparities: difference in the penetration of the coatings into the wood and drying shrinkage. Drying-shrinkage is an intrinsic property of inorganic materials in general and silicate-based materials such as concrete, but could not alone justify the high gap between the thickness values of the coatings. The fact that the adhesion strength is inversely related with the thickness suggests different levels of penetration of the coatings within the wood corresponding to various levels of contribution of the mechanical interlocking at the interface between the substrate and the coatings. The silica-to-alkali ratio and organosilane content modulate the particle size distribution in the dispersion, pH and the binding properties. The pH and adhesion strength of the coatings were found likely to be correlated following a polynomial law, but a high number of samples are required to validate the results. High pH could favor the penetration of the liquid coatings into the wood, reducing the final thickness and increasing the adhesion due to mechanical anchorage.

The morphological characterization of the coatings revealed the presence of microdefects (cracks, holes) throughout the coating layers. The defects probably resulted from the shrinkage induced by fast drying of the coatings as reported in many inorganic materials. Fast drying also limits the advancement of the curing as observed by lower adhesion values of the coatings cured in ambient conditions (low RH) compared to the coatings cured in a climate room (high RH).

The addition of potassium methylsiliconate, an organosilane-based compound, has increased the contact angle of water to more than 100° on the silicate coatings, but the benefit on water permeability was not noticeable. The application of the coatings by using a two-step brushing as performed in this work has not solved this drawback already reported in a previous work for a similar coating which was applied only by a single run with a standard coating’s applicator. The presence of open pores emerging through the surface probably exacerbates the water permeability of the coatings despite a certain level of hydrophobicity. However, the silicate coating with potassium methysiliconate (SW2) exhibited an improved adhesion value and reduced microdefects after immersion in water while the adhesion strengths of the other coatings were reduced, and macrocracks and debonding were observed. This result confirmed the ability of an organosilane-based compound to increase the flexibility of the coatings providing finishes capable of withstanding the dimensional changes of wood in some extent without further cracking. The increase of the adhesion strength of SW2 was attributed to additional curing reactions occurring during the drying process. The effect of methylsiliconate in the sol-silicate coatings was mitigated and could not be explained at this level of knowledge (insufficient data).

Most of the drawbacks highlighted in this work are expected to be overcome through an optimization of the coating properties. The control of rheological properties, such as levelling and the addition of compounds that are able to retain water and delay the drying of the coatings, can be useful to reduce formation of microcracks. The particle sizes of reactive additives, silica-to-alkali ratio of the binding phase and related particle sizes of the silicate species (monomer, oligomers to nanosilica), and the nature and amount of the organosilanes are parameters that could be optimized in the development of durable paints for wood. A solution to the problem of adhesion can be the utilization of primer coatings. The adhesion values of all the coatings were increased by the application of PVAc, or dextrin products containing colloidal silica.

## 4. Conclusions

Silicate and sol-silicate wood coatings were prepared in this study by changing the composition of the silicate binding phase from potassium silicate to mixtures containing potassium methysiliconate and colloidal silica. The coatings exhibited different behavior at the surface of beech wood including thickness and color, adhesion, and surface roughness. The sol-silicate coatings showed higher lightness, but lower adhesion in comparison to the silicate coatings. The yellow-red colors of the wood surface were almost completely concealed (a* and b* values between –1 and +1) attributed to good coverage ability of the coating paints. The adhesion strength values of the silicate coatings are quite adequate (above 3 MPa) for wood coatings, whereas the values for the sol-silicates were relatively low. The difference in the thickness of the coatings were attributed to the different level of penetration of the coatings in wood, and with adhesion were related to the pH of the liquid coatings. Microdefects were revealed within the coatings by optical microscopy and SEM. The coating roughness measured by CSLM was dependent on the magnification applied during the measurement and varied with the coating formulation. The arithmetic average roughness *S*_a_ was between 5 and 10 µm at magnification 5× and between 2.5 and 10 µm at magnification 20×. The maximum peak-to-valley height *S*_z_ was especially high at magnification 5× (between 100–170 µm) and above the measured coating thickness. The results suggested the presence of open porosity emerging through the coating’s surface. The open porosity and cracks are probably responsible for the water permeability of the coatings, particularly for the silicate coating containing methylsiliconate which exhibited a relatively high contact angle and low wettability. The resistance of this coating to further cracking under the action of water and even improvement of its mechanical adhesion after exposure to water and drying are interesting findings in the route of development of new coatings for wood.

The silicate coatings showed generally better adhesion than the sol-silicate coatings on wood without or with the mineralized primers investigated. The addition of an organosilane compound is promising for the durability of the silicate coatings; its use reduces cracking and debonding and loss of adhesion caused moisture and related dimensional changes. Cheap primers, such as polyvinyl acetate and dextrin-silica, could be used to increase the adhesion of the coatings and could potentially allow the utilization of the sol-silicate coatings. Further research efforts are required to reduce microcracking, holes, and open pores, and liquid water permeability.

## Figures and Tables

**Table 1:** Recipe for the preparation of the silicate and sol-silicate coatings. The values are weight percentages of the component in the coatings.

Binder	Sty-Acr	CaCO_3_	ZnO	Talc	TiO_2_	BD20	BQ40	BA11	HEC	DeF	Water
37.5	8	16	6	6	5	0.2	0.6	0.3	0.2	0.2	20

**Table 2:** Formulation of the binders and coating designations.

Binder	Formulation (wt %) ^a^ and Properties					Coating Designation ^b^
	KSi	MeKSi	Ludox^®^ AS 40	pH	Density (Kg m^−3^)	
Binder 1	100	/	/	12.13	1.31	SW1
Binder 2	75	25	/	12.41	1.28	SW2
Binder 3	25	/	75	11.49	1.26	SW3
Binder 4	/	25	75	12.26	1.26	SW4
Binder 5	/	15	85	11.91	1.24	SW5

^a^ Water was added in each composition to adjust the theoretical solid content to 30%. ^b^ SW1 and SW2 are silicate solutions and SW3, SW4, and SW5 sol-silicates.

**Table 3:** pH, density, and viscosity at different shear rate of the coatings.

Coating	pH (1 h)	pH (24 h)	Density (kg m^−3^) *	Viscosity at Various Shear Rate (Pa·s) *		
				0.1 s^−1^	1 s^−1^	1000 s^−1^
SW1	12.05	11.95	1.42	1119.5	90.5	0.16
SW2	11.91	11.84	1.41	632.7	62.3	0.23
SW3	11.42	11.26	1.41	1545.2	26.4	0.08
SW4	11.40	11.27	1.40	1557.4	91.8	0.05
SW5	11.07	10.91	1.41	850.9	10.6	0.16

* The values were obtained after 24 h of maturation.

**Table 4:** Surface CIELAB color space parameters of the coatings at the surface of beech wood (application rate 220 g·m^−2^).

Sample	L* ^‡^	a* ^‡^	b* ^‡^
Uncoated wood	74.41(0.99)	6.06(0.22)	16.89(0.33)
SW1	88.68(0.88)	0.23(0.13)	−1.03(0.25)
SW2	90.65(1.17)	−0.15(0.12)	−0.80(0.24)
SW3	92.85(0.15)	0.11(0.07)	0.36(0.05)
SW4	91.92(0.70)	0.07(0.12)	0.93(0.38)
SW5	92.50(0.40)	0.07(0.09)	1.00(0.06)

**^‡^** The values in parentheses are standard deviations.

## Data Availability

The raw data presented in this study are available on Zenodo via http://doi.org/10.5281/zenodo.4777893 accessed on 21 May 2021.

## Supplementary Materials

The following are available online at https://www.mdpi.com/article/10.3390/ma14133559/s1, The supplementary material submitted with the manuscript reports Figure S1: Photograph of the coatings at the surface of wood (application rate 220 kg m^−2^; CR curing); Figure S2: 3D micrographs at magnification 5× of a silicate coating SW1 (a,a’) and a sol-silicate SW4 (b,b’) illustrating the typical features of the coatings at this magnification; Figure S3: 2D micrographs of the surface of the coatings cured in CR and AC at magnification 5× (The scale bars represent 1 mm) and Figure S4: Comparison of Sa and Sz surface roughness parameters of the coatings cured in CR and AC at magnification 5×. CR: Climate room ((23 ± 3) °C and (75 ± 2)% relative humidity)); AC: ambient conditions ((23 ± 3) °C and (25 ± 5)% relative humidity)).
