# Supplementary material for: Preparation, Surface Characterization, and Water Resistance of Silicate and Sol-Silicate Inorganic–Organic Hybrid Dispersion Coatings for Wood
Source: Materials (Basel). 2021 Jun 25;14(13):3559. doi: 10.3390/ma14133559 (PMC8269507; doi:10.3390/ma14133559)
Supplement: Supplementary file 1 [file materials-14-03559-s001.zip › materials-1249680-SM.pdf]

materials

MDPI

Article

Preparation, Surface Characterization, and Water Resistance of Silicate and Sol-Silicate Inorganic–Organic Hybrid Dispersion Coatings for Wood

Arnaud Maxime Cheumani Yona [1,2,*], Jure Žigon [1], Alexis Ngueteu Kamlo [2], Matjaž Pavlič [1], Sebastian Dahle [1], and Marko Petrič [1]

[1] Department of Wood Science and Technology, Biotechnical Faculty, University of Ljubljana, Jamnikarjeva ulica 101, SI-1000 Ljubljana, Slovenia; jure.zigon@bf.uni-lj.si (J.Ž.); Matjaz.Pavlic@bf.uni-lj.si (M.P.); Sebastian.Dahle@bf.uni-lj.si (S.D.); marko.petric@bf.uni-lj.si (M.P.)

[2] Macromolecular Research Team, Faculty of Science, University of Yaoundé 1, Yaoundé P.O. 812, Cameroon; akngueteu@yahoo.fr

* Correspondence: ArnaudMaximeCheumani.Yona@bf.uni-lj.si

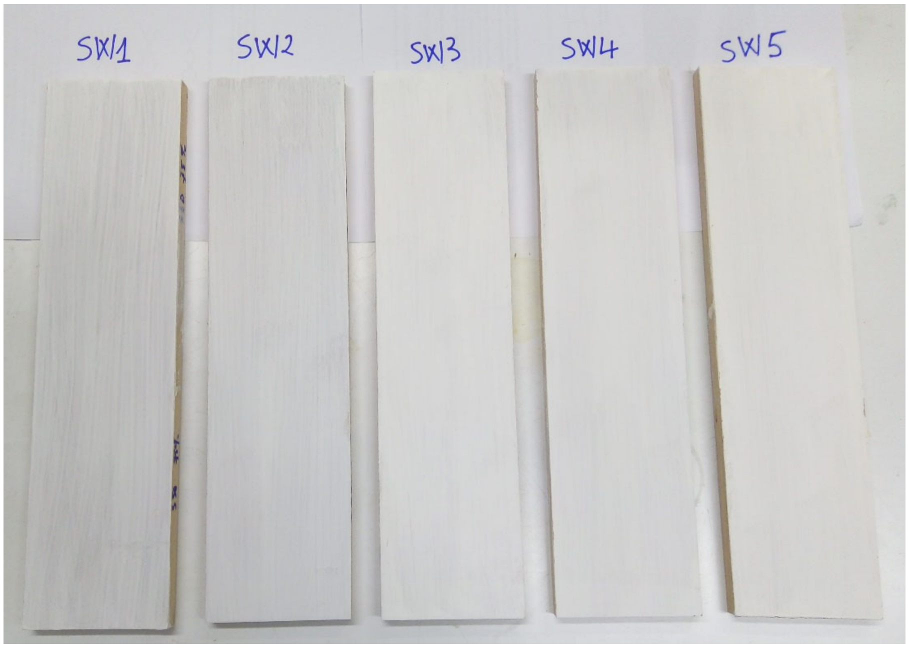

Figure S1. A photograph of the coatings at the surface of wood (application rate 220 kg m^{-2}).

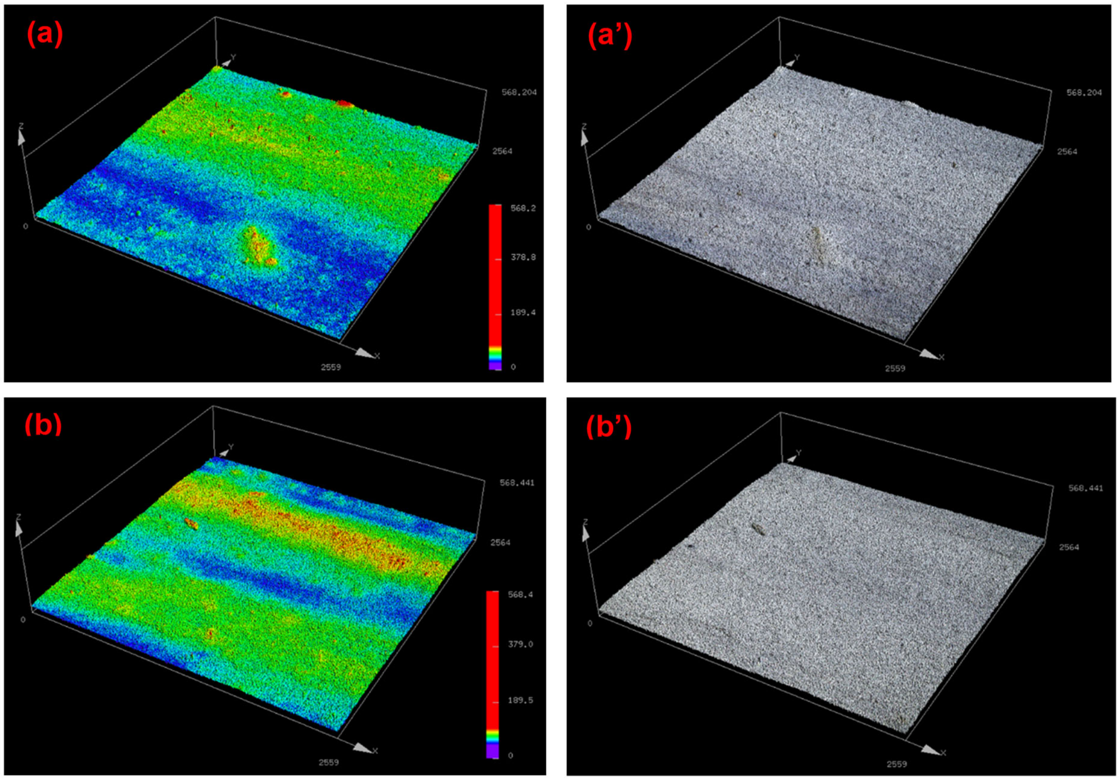

Figure S2. 3D micrographs at magnification 5× of a silicate coating SW1 (a, a') and a sol-silicate SW4 (b, b') illustrating typical features of the coatings at this magnification.

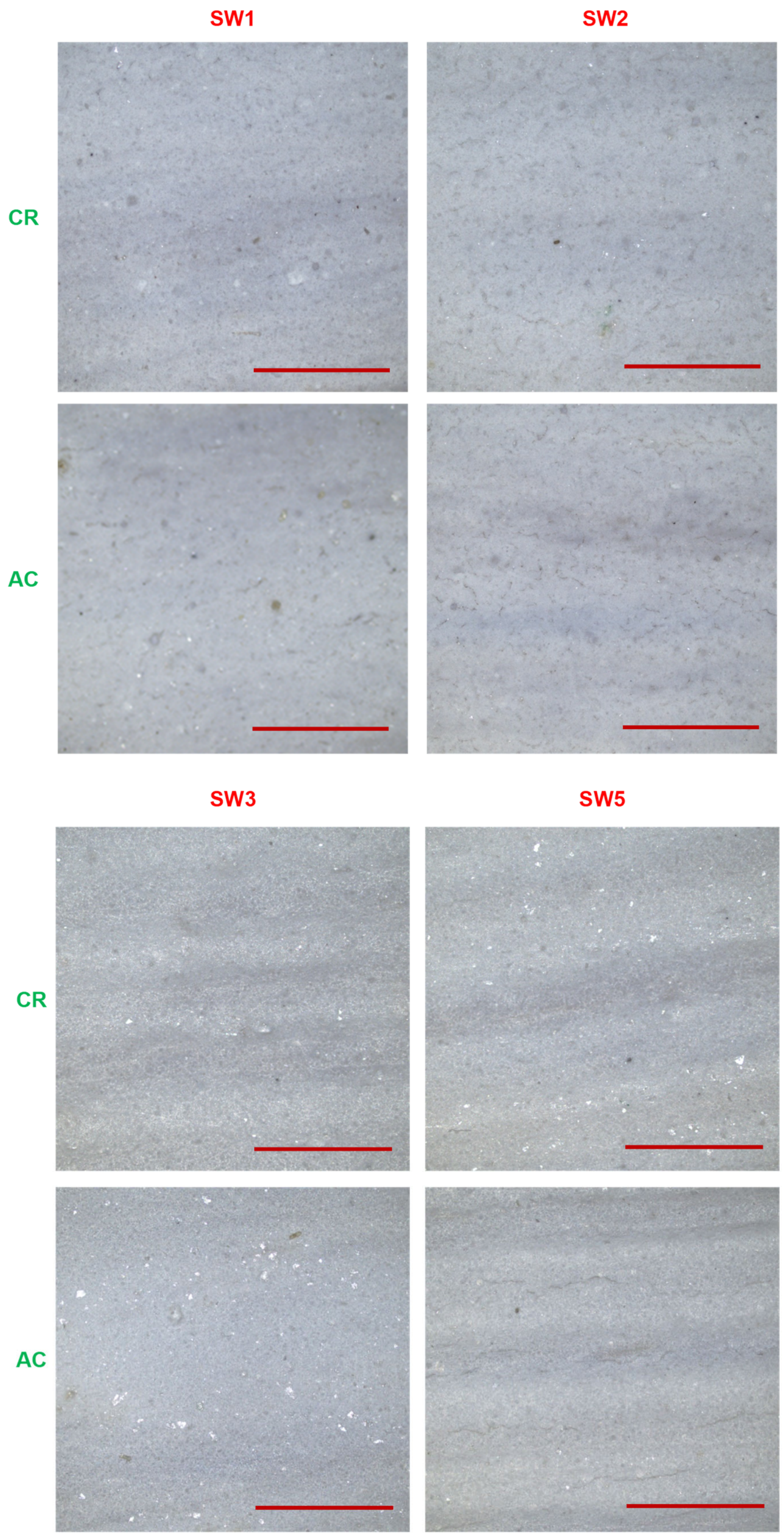

Figure S3. 2D micrographs of the surface of the coatings cured in CR and AC at magnification 5× (The scale bars represent 1 mm). CR: Climate room ((23±3) °C and (75±2) % relative humidity)); AC: ambient conditions ((23±3) °C and (25±5) % relative humidity)).

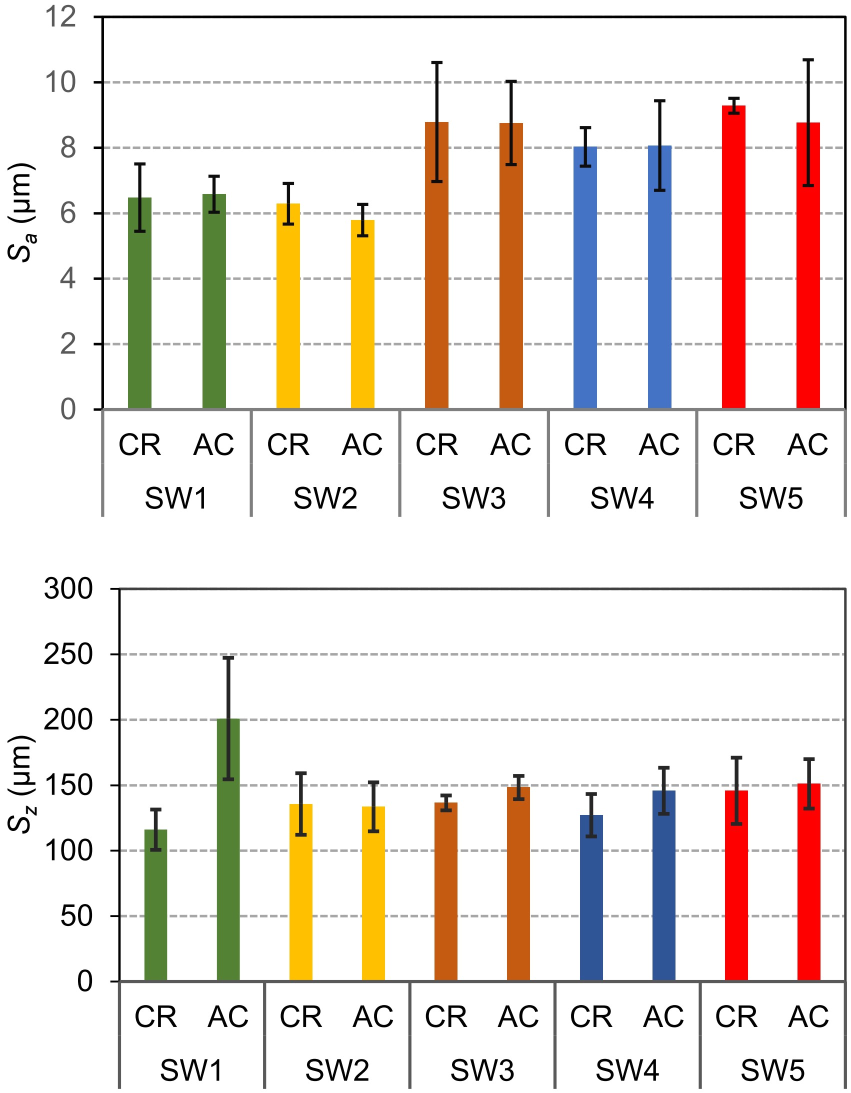

Figure S4. Comparison of Sa and Sz surface roughness parameters of the coatings cured in CR and AC at magnification 5×. CR: Climate room ((23±3) °C and (75±2) % relative humidity)); AC: ambient conditions ((23±3) °C and (25±5) % relative humidity)).
